# Impact of restenting for recurrent colonic obstruction due to tumour ingrowth

**DOI:** 10.1002/bjs5.34

**Published:** 2018-03-15

**Authors:** L. Clarke, H. Abbott, P. Sharma, T. W. Eglinton, F. A. Frizelle

**Affiliations:** ^1^ Colorectal Unit, Christchurch Hospital Christchurch New Zealand; ^2^ Department of Surgery, University of Otago Christchurch New Zealand

## Abstract

**Background:**

Endoscopic stenting is used to palliate malignant large bowel obstruction. A proportion of patients will develop recurrent obstruction due to tumour ingrowth and require reintervention. This study aimed to assess the outcome (clinical success and complication rates) of endoscopic reintervention compared with surgical intervention in patients with stent obstruction due to tumour ingrowth.

**Methods:**

This was an observational study using data from a database of patients who underwent palliative colonic stenting between January 1998 and March 2017 at Christchurch Public Hospital.

**Results:**

A total of 190 patients underwent colonic stent insertion, for palliation in 182 cases. Reintervention was performed in 55 (30·2 per cent). Thirty‐one patients (17·0 per cent) developed obstruction within the stent at a median of 4·6 (i.q.r. 2·3–7·7) months after the procedure. Of these, 21 had endoscopic restenting and ten underwent surgery. Restenting had technical and clinical success rates of 100 per cent, and involved a significantly shorter length of stay compared with surgery (median 2 (i.q.r. 1–4) versus 11 (6–19) days respectively; P = 0·006). Seven of the 21 patients in the restented group underwent a third palliative intervention. The overall stoma rate in the restented group was significantly lower than that in the surgical group (4 of 21 versus 10 of 10; P < 0·001). There was no difference in complications or survival between the two groups.

**Conclusion:**

Among palliative patients who develop malignant stent obstruction, endoscopic restenting had a high chance of technical success. It resulted in a shorter hospital stay and lower stoma rate than those seen after surgery.

## Introduction

Colonic stenting has become popular for palliating large bowel obstruction in patients with incurable disease, in the hope of avoiding surgery and stoma formation^1^. Earlier studies^2^ ^3^ on stenting suggested a high rate of perforation, although more recent series^4^, ^5^, ^6^, ^7^ have demonstrated an acceptably low level of complications and high clinical success rates. Compared with palliative surgery, stenting has a lower clinical success rate but a shorter stay in hospital, a reduction in complications, reduced stoma formation, earlier commencement of chemotherapy and improved quality of life^8^, ^9^, ^10^, ^11^, ^12^, ^13^, ^14^.

As a result of advances in chemotherapy, the life expectancy of patients with metastatic colorectal cancer is increasing^15^; the median survival of patients after colonic stenting is 7–20 months^4^ ^7^, ^16^ ^17^. Some 1–17 per cent of patients with a stent develop recurrent obstruction due to tumour ingrowth^5^ ^17^, ^18^, ^19^. The options for further treatment at this stage are either surgery or reinsertion of a second stent within the first. The aim of this study was to examine the success and complication rates of endoscopic reintervention for stent obstruction, and to compare these outcomes with those for surgical intervention.

## Methods

This was an observational study using data from a dedicated registry of patients who had undergone colonic stent insertion for malignant large bowel obstruction between 1998 and 2017 at Christchurch Public Hospital. Ethical approval was obtained from the Upper South B Regional Ethics Committee.

Patients who had successful primary stent insertion and subsequently underwent intervention for large bowel obstruction at the site of an existing stent were identified from this database. The clinical records of these patients were then examined. From this group those patients who underwent a second attempted stent placement (restented group) were compared with those who had a surgical procedure (surgery group).

### Inclusion and exclusion criteria

The medical records of all patients with a colonic stent *in situ* who underwent surgical or endoscopic reintervention were examined. Patients who had surgery for early complications, such as perforation after the first stent placement, were excluded. Those who had stent migration and subsequent reobstruction were also excluded, as were patients who underwent curative resection following ‘bridge to surgery’ stenting. Patients who had obstruction secondary to faecal impaction managed conservatively were also excluded. Patients who had reintervention for large bowel obstruction due to tumour ingrowth during the study period were included.

### Interventions

The urgency of intervention was defined as acute if the patient presented as an emergency with clinical and radiological evidence of complete colonic obstruction. Elective stents were performed on a planned list. All patients had CT to confirm the diagnosis before the intervention. The choice of surgical or endoscopic intervention was at the treating surgeon's discretion.

The technique of stent insertion has been described previously^4^. Briefly, under light sedation or general anaesthetic the endoscope was introduced to the point of obstruction. A Jagwire™ Super Stiff guidewire (Boston Scientific, Natick, Massachusetts, USA) was then deployed across the stricture, a tapered‐tip Tandem™ XL catheter (Boston Scientific) was introduced over this, and contrast was injected to confirm intraluminal position under fluoroscopy. The type of stent chosen was at the endoscopist's discretion, and was deployed under direct endoscopic vision with fluoroscopy.

### Definitions

For patients undergoing placement of a second stent (stent within a stent), technical success was defined as the ability to deploy a stent across the obstruction. Clinical success was defined as resolution of obstructive symptoms, demonstrated by passage of stool or flatus, within 24 h.

Data were collected on success rates, complications and length of stay. Reintervention rates and the need for a stoma were also recorded. Survival was calculated from the date of reintervention.

### Statistical analysis

Data were recorded in a purpose‐designed Microsoft® Access® (Microsoft, Redmond, Washington, USA) database. The restented group was analysed and then compared with the surgery group. Fisher's exact test was used to assess the independence of categorical data, and the Mann–Whitney *U* test for continuous data. Significance was set at *P* < 0·050.

## Results

**Figure 1:**
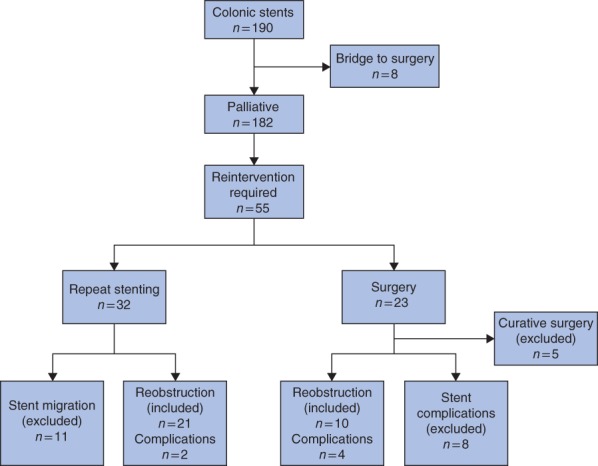
Flow chart of patients undergoing reintervention after colonic stenting

A total of 190 patients underwent colonic stent insertion over the study period. Of these, eight procedures were performed as a bridge to surgery and were excluded. Of the remaining 182 patients, 55 (30·2 per cent) underwent a reintervention: 32 had repeat endoscopic stent insertion and 23 had surgery. The indications for reintervention are shown in *Fig*. 1. Eight had surgery for complications of their initial stent and were excluded. Five had an excellent response to chemotherapy with subsequent change of treatment aim from palliative to curative, and resection of the primary tumour. They were included in the bridge to surgery group and excluded from further analysis. Of the patients undergoing endoscopic reintervention, 11 had migration of the primary stent and went on to develop recurrent obstruction at a median of 2·9 (i.q.r. 1·3–5·3) months. As these were not obstructions within a stent, they were also excluded from analysis. This left 31 patients (17·0 per cent) who developed reobstruction within a stent at a median of 4·6 (i.q.r. 2·3–7·7) months. Of these, 21 underwent endoscopic restenting (restented group) and ten had surgical intervention (surgery group).

There were no significant differences in age, sex, ASA grade, tumour site or site of metastatic disease between the two groups (*Table* 1). Two of the ten patients in the surgery group had a gynaecological malignancy; all other patients had primary colorectal cancer.

**Table 1:** Demographic and clinical characteristics

	Restented group (*n* = 21)	Surgery group (*n* = 10)	*P* †
Age (years)*	65 (32–87)	68 (32–93)	0·876‡
Sex ratio (M : F)	14 : 7	4 : 6	0·247
ASA fitness grade			0·910
I	2	0	
II	4	0	
III	9	7	
IV	2	1	
Missing	4	2	
Cancer type			
Colorectal	21	8	0·098
Other	0	2	
Tumour site			0·671
Right	1	0	
Left	18	8	
Rectal	2	2	
Metastasis			
Liver	18	5	0·026
Lung	4	3	0·652
Peritoneal	6	2	0·690
Urgency			
Acute	19	7	0·296
Elective	2	3	
Chemotherapy	20	9	1

* Values are mean (range).

† Fisher's exact test, except

‡ Mann–Whitney *U* test.

There was no significant difference in the urgency of the interventions between the two groups, in the proportion of patients receiving chemotherapy, or the time to reintervention (*Table* 2).

**Table 2:** Comparison of outcomes in restented and surgery groups

	Restented group (*n* = 21)	Surgery group (*n* = 10)	*P* †
Time from first stent to reintervention (months)*	4·6 (2·8–7·6)	4·3 (2·0–8·6)	0·568‡
Length of postprocedure stay (days)*	2 (1–4)	11 (6–19)	0·006‡
Postprocedural complications	2	4	0·067
Stoma	4	10	< 0·001
Reintervention	7	1	0·222
Readmission	7	3	1·000
30‐day mortality	0	0	1·000
1‐year survival	4	2	1·000
2‐year survival	2	1	1·000
Time from intervention to death (months)*	5·1 (2·5–9·9)	7·3 (2·9–14·8)	0·881‡

* Values are median (i.q.r.).

† Fisher's exact test, except

‡ Mann–Whitney *U* test.

Technical and clinical success rates were 100 per cent in the restented group. All patients in the surgery group had creation of a stoma, resulting in relief of the obstruction.

### Length of stay and complications

Patients in the restented group had a significantly shorter postprocedural stay in hospital compared with those undergoing surgery (median 2 (i.q.r 1–4) *versus* 11 (16–19) days respectively; *P* = 0·006).

There were two complications in the restented group. One patient developed atrial flutter in the evening after stenting and was commenced on a beta‐blocker. There was no clinical evidence of colonic ischaemia and the patient was discharged 2 days after the procedure. The second patient had chest pain and a raised troponin level. This was managed medically and the patient was discharged 8 days postprocedure.

There were four complications in the surgery group, two of which occurred within 30 days. One patient who underwent laparoscopic formation of a loop sigmoid colostomy developed a retracted stoma resulting in a Hartmann's procedure with formation of an end colostomy. One patient was readmitted 7 days after discharge with dehydration due to a high‐output ileostomy; intravenous rehydration and correction of electrolyte imbalance was required. Two patients had complications after 30 days, one with multiple admissions due to stomal prolapse and the other developed recurrent bowel obstruction after a loop transverse colostomy for gynaecological malignancy with peritoneal metastases. This resolved with non‐operative management.

There was no difference in readmission rates between the restented and surgery groups (7 of 21 *versus* 3 of 10 respectively).

Of 21 patients in the restented group, seven had a further (third) intervention (at a median of 2 months), compared with one of the ten patients in the surgery group (*P* = 0·222). In the restented group, one patient had a third stent inserted endoscopically and six patients underwent surgery. The overall stoma rate was 19 per cent (4 of 21) in the endoscopic reintervention group and 100 per cent (10 of 10) in the surgery group (*Table* 2).

### Survival

No patient in either group died within 30 days of the procedure. There was no difference in 1‐ and 2‐year survival between the groups. Only two of 21 restented patients and one of ten patients in the surgery group were alive at 2 years after the reintervention. The median time to death was 5·1 (i.q.r. 2·5–9·9) months in the restented group and 7·3 (2·9–14·8) months after surgery.

There was no statistically significant difference in median survival between those who underwent a third intervention and those who did not: 8·7 (3·4–27·1) *versus* 4·8 (1·9–8·1) months (*P* = 0·177).

## Discussion

This study demonstrated that 17·0 per cent of patients who had successful palliative colonic stent placement underwent reintervention for recurrent obstruction at the site of the stent. This rate is comparable to findings from other series^5^ ^15^, ^19^ ^20^. The median time to reintervention in this study was 4·6 (i.q.r. 2·3–7·7) months, similar to previous reports^16^ ^18^. This duration of patency should be taken into account when selecting the appropriate management in patients with stage IV colorectal cancer who present with large bowel obstruction.

A technical and clinical success rate of 100 per cent for repeat stenting was achieved in this small group, which compares favourably with series of primary stenting 5 7, 21. The morbidity rate following the procedure was low, and managed without surgical or radiological intervention. There were no early deaths, suggesting that repeat stenting is no more difficult than a primary procedure.

The median length of postprocedure stay was significantly shorter in patients undergoing repeat stenting than in those who had surgery (2 *versus* 11 days; *P* = 0·006). This might be important, as the median time to death in this group of patients was only 6 months. However, the study did not examine total recovery time or health‐related quality of life.

Overall, one‐third of patients undergoing repeat stenting required a further reintervention, at a median of 2 months. This did not reflect increased survival, and possible explanations include differences in tumour biology leading to faster recurrence or inability of the stent to expand fully within the constraints of a previously inserted one. Despite the higher rate of reintervention in the restented group, only four of 21 patients ended up with a stoma, compared with all patients in the surgery group. This confirms earlier findings that colonic stenting for large bowel obstruction reduces the rate of stoma formation^21^. Stomas are associated with morbidity, including hernia formation and peristomal skin irritation^22^, ^23^, ^24^. In this small study, two of the ten patients undergoing surgery and stoma formation had subsequent complications from their stoma, and one required a revision. Quality of life is recognized as the most important factor in palliative surgery^25^, and the impact of stomas on quality of life has been well described^9^ ^10^. In the present study, 17 (81 per cent) of the 21 patients undergoing colonic restenting could be palliated successfully, without a stoma, until their death, supporting the role of repeat stenting in the context of palliative care.

Weaknesses of this study include its observational nature as well as the small sample size. The choice of repeat stenting or surgery was at the surgeon's discretion, inviting selection bias where patients with a lower disease burden undergo surgery. There was, however, no difference in distribution of metastases, ASA grade or survival between the groups, suggesting comparability. Other factors such as tumour location, technical considerations and surgeon expertise would have influenced the choice of intervention, and these were not recorded in this study.

Repeat colonic stenting has a good success rate with low rates of complications. Although one‐third of these patients will require further reintervention, about 80 per cent of patients undergoing stenting can be palliated successfully endoscopically without requiring a stoma.

## Disclosure

The authors declare no conflict of interest.
